# Consensus and uncertainty in the geographic range of *Aedes aegypti* and *Aedes albopictus* in the contiguous United States: Multi-model assessment and synthesis

**DOI:** 10.1371/journal.pcbi.1007369

**Published:** 2019-10-10

**Authors:** Andrew J. Monaghan, Rebecca J. Eisen, Lars Eisen, Janet McAllister, Harry M. Savage, John-Paul Mutebi, Michael A. Johansson

**Affiliations:** 1 Research Computing, University of Colorado Boulder, Boulder, Colorado, United States of America; 2 Division of Vector-Borne Diseases, Centers for Disease Control and Prevention, Fort Collins, Colorado, United States of America; 3 Division of Vector-Borne Diseases, Centers for Disease Control and Prevention, San Juan, Puerto Rico, United States of America; Yale School of Public Health, UNITED STATES

## Abstract

*Aedes (Stegomyia) aegypti* (L.) and *Ae*. *(Stegomyia) albopictus* (Skuse) mosquitoes can transmit dengue, chikungunya, yellow fever, and Zika viruses. Limited surveillance has led to uncertainty regarding the geographic ranges of these vectors globally, and particularly in regions at the present-day margins of habitat suitability such as the contiguous United States. Empirical habitat suitability models based on environmental conditions can augment surveillance gaps to describe the estimated potential species ranges, but model accuracy is unclear. We identified previously published regional and global habitat suitability models for *Ae*. *aegypti* (n = 6) and *Ae*. *albopictus* (n = 8) for which adequate information was available to reproduce the models for the contiguous U.S. Using a training subset of recently updated county-level surveillance records of *Ae*. *aegypti* and *Ae*. *albopictus* and records of counties conducting surveillance, we constructed accuracy-weighted, probabilistic ensemble models from these base models. To assess accuracy and uncertainty we compared individual and ensemble model predictions of species presence or absence to both training and testing data. The ensemble models were among the most accurate and also provided calibrated probabilities of presence for each species. The quantitative probabilistic framework enabled identification of areas with high uncertainty and model bias across the U.S. where improved models or additional data could be most beneficial. The results may be of immediate utility for counties considering surveillance and control programs for *Ae*. *aegypti* and *Ae*. *albopictus*. Moreover, the assessment framework can drive future efforts to provide validated quantitative estimates to support these programs at local, national, and international scales.

## Author summary

*Aedes aegypti* and *Ae*. *albopictus* mosquitoes can transmit dengue, chikungunya, yellow fever, and Zika viruses, yet because of limited data the edges of the geographic range of these important species remain uncertain. We assessed numerous previously published model-based estimates of the range of these mosquitoes in the United States and combined those models to produce calibrated estimates of the probability of finding each mosquito in each county. Comparing these estimates to county-level data, we found that there are areas of substantial uncertainty and specific areas where model-based predictions do not align well with available data. The results provide specific information that can help guide national- or state-level efforts to monitor and control *Ae*. *aegypti* and *Ae*. *albopictus*. Beyond the specific findings, this approach to leveraging limited data and multiple quantitative models can be employed in other settings to better characterize the distribution of these species and other medically important vectors globally.

## Introduction

*Aedes (Stegomyia) aegypti* (L.) and *Ae*. *(Stegomyia) albopictus* (Skuse) mosquitoes are best known for their impact on human health as vectors of dengue, chikungunya, yellow fever, and Zika viruses. These viral pathogens are of growing concern due to the recent chikungunya and Zika pandemics [1–3], the increased burden of dengue in recent decades [4,5], and continued yellow fever outbreaks in Africa and South America [6,7]. Risk of acquiring these viruses, especially dengue virus, is common throughout the tropics and subtropics where *Ae*. *aegypti* or *Ae*. *albopictus* are common [8]. In temperate regions, however, the distribution of these vectors is dynamic and not well defined [8,9], posing a challenge for public health officials trying to address the risk of arbovirus introduction.

Risk of arbovirus invasion in areas where the mosquitoes may be present has led to an extensive body of research leveraging existing data to empirically estimate the global or regional geographic distributions of suitable environmental conditions that would support the establishment of *Ae*. *aegypti* and *Ae*. *albopictus* [10–30]. This research has employed numerous analytical approaches including: genetic algorithms [31]; random forest models [32]; boosted regression trees [33]; maximum entropy models [34]; generalized linear models [35]; alpha-shapes models [20,36]; general climate modeling tools [37,38]; and other ecological niche models (e.g., [11,28]).

However, despite the importance of the problem and breadth of research to address it, fundamental challenges remain. First, large-scale mosquito surveillance data are mostly limited to presence records. This is an intrinsic challenge; finding a mosquito where a species is abundant is generally straightforward, but proving that a species does not occur somewhere is much more difficult (this is a particularly vexing problem for invasive species that may be absent from areas that are environmentally suitable). Modeling approaches aiming to distinguish presence and absence therefore rely on pseudo-absence points that represent locations where the species is likely absent. Models often assume that all locations without documented presence are pseudo-absence locations [30], but other approaches use a subset of these that are not similar to presence locations in other measurable characteristics [25]. Because true absence locations are unknown, especially at the margins of species distributions, metrics of the accuracy of estimated distributions, such as specificity, positive predictive value (PPV), and negative predictive value (NPV), are difficult to assess.

A second challenge is that even presence data are sparse in space and time, making it difficult to withhold some of the data for an out-of-sample assessment of model accuracy. Thus, the main accuracy metric for models is how well they fit the specific presence data used to generate them, which is not an assessment of their ability to predict. This issue also makes it difficult to objectively compare the accuracy among multiple models. Without out-of-sample testing, even consensus across multiple approaches is not necessarily a good indicator of presence or absence.

Here we addressed these challenges in the context of the geographic distribution of *Ae*. *aegypti* and *Ae*. *albopictus* in the contiguous United States (CONUS), where the two species have a long, dynamic, and intertwined history. We focused on this region because it is at the northern geographic margin of suitability for *Aedes* mosquitoes in the Western Hemisphere [39,40], is relatively data rich, has been the focus of numerous previous studies, and is a region with risk of *Aedes*-transmitted arbovirus transmission yet substantial uncertainty regarding the distributions of both species. While *Aedes*-transmitted arboviruses are less common in CONUS than in tropical areas, infected travelers frequently introduce these viruses [41–44] and identifying the range of these mosquitoes is key to assessing the risk of autochthonous transmission.

Similar to other locations, the distributions of *Ae*. *aegypti* and *Ae*. *albopictus* in CONUS are dynamic and surveillance data are limited [40,45]. *Aedes aegypti* has likely been in the U.S. for nearly 400 years according to historical accounts of dengue and yellow fever [39,46], but mosquito surveillance records collected since the mid-1990s from at least 291 counties suggest that the range of *Ae*. *aegypti* continues to change, with ongoing reestablishment or expansion in some counties [40,45,47]. The first established *Aedes albopictus* population in the U.S. was documented in 1985 in Texas [48] and the mosquito has since been recorded in at least 1,568 counties [45]. Invading *Ae*. *albopictus* also appear to have displaced *Ae*. *aegypti* in some locations through inter-specific competition [49–51].

We aimed to synthesize and assess existing model estimates for the geographic distributions of these two species in CONUS using novel approaches to evaluate and combine information from multiple habitat suitability models. First, we collected or recreated published global or U.S.-specific estimates of habitat suitability for both species. We then evaluated these estimates for all counties in the contiguous U.S. using the most recent and comprehensive presence records available [40,45] and pseudo-absence classifications for counties where surveillance for mosquitoes has taken place, representing data that have not previously been used to either develop or evaluate models. Next, we used accuracy metrics to develop accuracy-weighted probabilistic ensemble species distribution estimates and identify key areas where estimates have low accuracy due to either uncertainty or bias. In summary, our objective was (for both species) to quantitatively assess current models of the geographic distributions, develop calibrated estimates for the probability of presence, and identify areas in which uncertainty is highest.

## Materials and methods

### Mosquito presence/absence records

For presence data, we used recently published county level *Ae*. *aegypti* and *Ae*. *albopictus* occurrence records from 1995–2016 [40,45], and historical records back to 1960 compiled from multiple sources [30]. *Aedes aegypti* or *Ae*. *albopictus* were considered “present” in a county if at least one mosquito of any life stage was collected and reported. Out of 3,111 counties in the contiguous U.S., 291 (*Ae*. *aegypti*) and 1,568 (*Ae*. *albopictus*) met this condition for presence. Additional information on the compilation of the occurrence data from 1995–2016 can be found in Hahn et al. [40,45].

**Fig 1:**
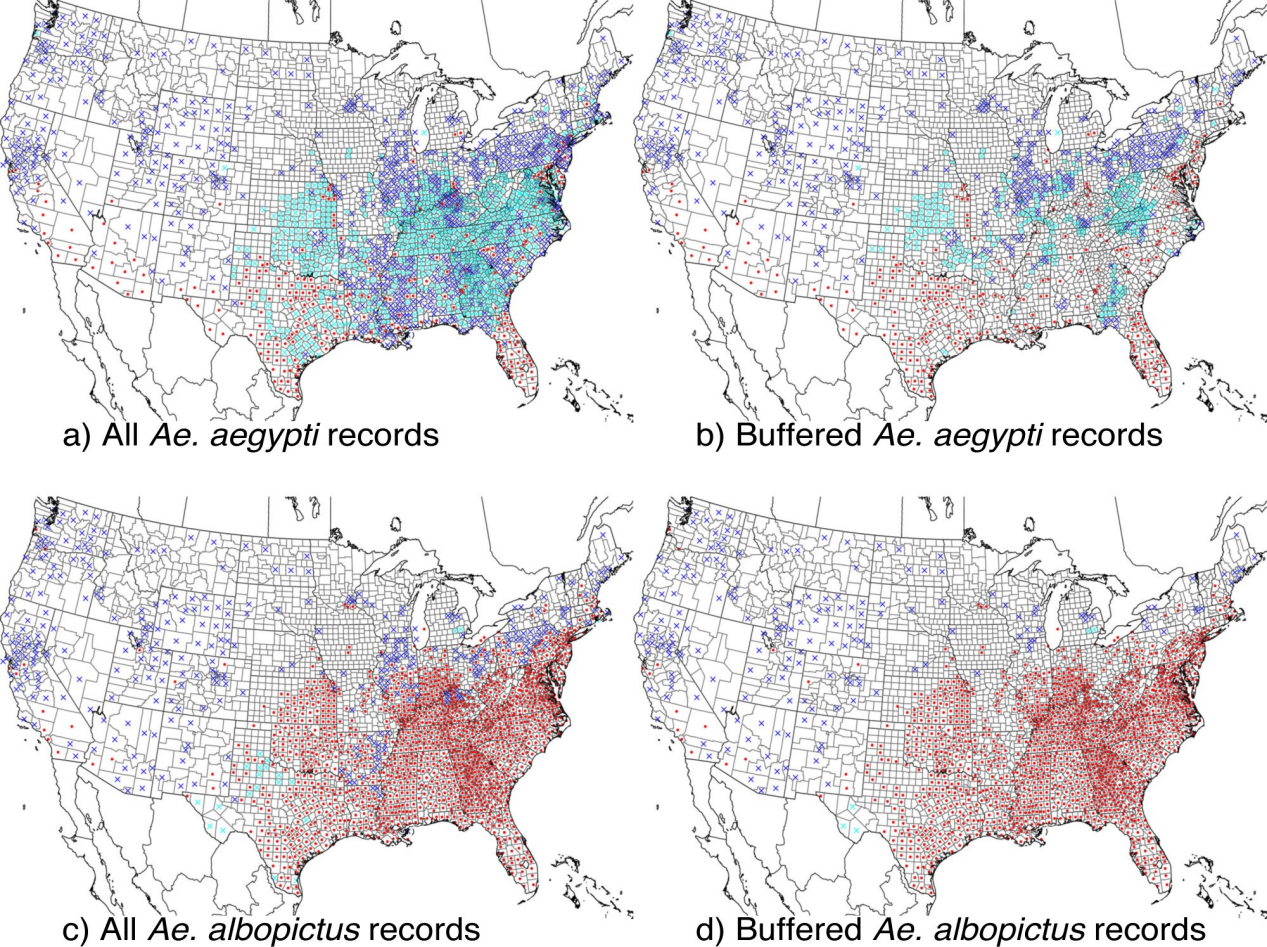
County-level presence or pseudo-absence of *Ae*. *aegypti* and *Ae*. *albopictus* in the contiguous U.S. (a) Counties with presence and pseudo-absence records for *Ae*. *aegypti* and (b) with 100 km buffered pseudo-absence. (c) *Ae*. *albopictus* unbuffered and (d) buffered presence and pseudo-absence. Red dots are presence records. Dark blue "x" symbols are pseudo-absence records based on counties with known vector surveillance. Light blue "x" symbols are additional pseudo-absence records based on counties in which the other species was reported but the species in question was not.

Counties where the species are absent are more difficult to define as ruling out the possibility that a species is present would require extremely intense and comprehensive surveillance. Previous work has estimated the absence of a species based on all counties without presence records for a given species [30]. Given the paucity of presence records in areas that are likely environmentally suitable, particularly for *Ae*. *aegypti*, this approach may penalize models for predicting suitability in counties where little surveillance has been conducted and the mosquito may actually be present. In developing our consensus models, we therefore assessed two additional indicators of absence to identify counties where detecting a species may have been more likely but the species still had not been reported. We defined these counties in which absence is more likely (but still unconfirmed) as “pseudo-absence” counties. First, we assumed that detection of either species would be equally probable if vector surveillance was implemented in a county. We therefore classified counties where species A had been reported, but not species B (as of 2017), as absent for species B. Second, we identified counties conducting full or partial surveillance for mosquitoes as of 2017 (compiled by JM from multiple sources, S1 Fig). We therefore limit absence counties to those which had surveillance but had not reported *Aedes* mosquitoes. Additionally, we assumed that counties without occurrence records which neighbor counties with occurrence records were also likely to have the species. To implement this assumption, we created a buffer of 100km around the centroid of each ‘occurrence’ county (we found that the centroid of the nearest neighbor county is within 100km for 99% of U.S. counties). We then excluded counties falling within that buffer from being classified as absent. Therefore, we categorized counties as “pseudo-absence” for species A only if there were no local reports of species A, there was evidence of mosquito surveillance or local reports of species B, and the county was at least 100km from the nearest county with reported species A. The original and refined pseudo-absence counties for each species are shown in Fig 1.

For model development we constructed a training (in-sample) dataset consisting of a random sample of 80% of the presence records and 80% of the pseudo-absence records. The remaining 20% of presence and pseudo-absence records were reserved as a testing dataset for independent (out-of-sample) model evaluation. This approach follows the methodology for evaluating predictive machine learning models [52].

### Identification of candidate models

PubMed and Google Scholar were used to identify global, Europe- or CONUS-specific empirical habitat suitability modeling studies for either species published since 1960. Each study was characterized by the species modeled, region of interest, model type (as classified in the introduction), climatic explanatory variables, non-climatic explanatory variables, outcome variable, time period of interest and spatial resolution.

From this inventory, we selected all models with either a digital version of the suitability map available or sufficient detail about data and methods to reproduce the map. Models published since 2012 (five years before we initiated this analysis) that were not already available electronically or readily reproducible, were requested from the authors. The models available electronically included Caphina et al. [20] (*Ae*. *aegypti*), Campbell et al. [24] (*Ae*. *aegypti* and *Ae*. *albopictus*), Kraemer et al. [25] (*Ae*. *aegypti* and *Ae*. *albopictus*), Monaghan et al. [28] (*Ae*. *aegypti*), Proestos et al. [26] (*Ae*. *albopictus*). and Johnson et al. [30] (*Ae*. *aegypti* and *Ae*. *albopictus*). For those models requiring reproduction we used the methods described in the original papers and climatic inputs derived from version 1.4 of Worldclim, a gridded monthly global climatology of near-surface temperature and precipitation representing historical conditions for 1950–2000 [53]. The models requiring reproduction were those of Christophers [54] (*Ae*. *aegypti*), Kobayashi et al. [11] (*Ae*. *albopictus*), Medlock et al. [12] (*Ae*. *albopictus*), the European Centre for Disease Prevention and Control [55] (*Ae*. *albopictus*), and Mogi et al. [18] (*Ae*. *albopictus*). An additional model produced by Caminade et al. [17] was not incorporated here because the minimum suitability condition for *Ae*. *albopictus* was equivalent to that of the previous model by Medlock et al. [12]. A summary of the candidate models with additional details can be found in the Supporting Information (S1 and S2 Tables).

### Model synthesis

All candidate models were produced as rasters and converted to county level maps using the raster grid cell nearest the centroid of each county. No adjustment was made for the temporal period over which each model was developed. Model outcomes were expressed as provided in the original models, either as continuous probability scores for presence (between 0 and 1) or as binary classifications (absence or presence). To facilitate analysis across all models, those with probabilistic predictions [17] were converted to binary scores. To do this, we first computed model sensitivity and specificity for each 0.01 increment of probability using the 80% training dataset for each species. We selected a cutoff probability by maximizing the sum of sensitivity and specificity, and dichotomized the results into presence/absence values for scores above/below this value (S2 Fig). We compared predictions with common binary outcome metrics using the training data: accuracy (the probability the model will correctly categorize counties); sensitivity (the proportion of counties with reported vectors that were estimated to be positive for the vector); specificity (the proportion of counties classified as pseudo-absent for vectors that were correctly estimated to be negative for the vector); positive predictive value (PPV; the proportion of counties estimated to be positive in which the vector had been reported) and the negative predictive value (NPV; the proportion of counties estimated to be negative which were classified as pseudo-absent counties for the vector). Note that all of these metrics included only counties classified as present or pseudo-absent for each species in the training data.

We then generated ensemble models for each mosquito species by replacing county-level positive and negative predictions from each candidate model with their in-sample PPV and 1-NPV values, respectively. Thus, each county in each candidate model was assigned a value reflecting the probability of being a true positive based on the county-specific prediction weighted by the model-specific performance. Averaging these values across the candidate models therefore produced accuracy-weighted ensemble predictions. We then used a binomial generalized additive model to calibrate the accuracy-weighted ensemble predictions to the training data such that the final county-level prediction is a calibrated probability of presence based on the suitability models and the presence/absence data.

### Ensemble model evaluation

The resulting species-specific ensemble models were evaluated in four ways. First, we assessed model calibration by binning predictions within deciles (0–0.1, 0.1–0.2, etc.) and comparing predictions to the proportion of counties classified as present within each bin (we used an exact binomial test to calculate a 95% confidence interval based on the number of counties falling in each bin). We assessed calibration separately for the training and testing datasets. Second, we dichotomized the ensemble model using a cutoff probability of 0.5 and assessed binary predictions of presence or absence for all models (candidate and ensemble) on the 20% testing dataset.

Third, we assessed ensemble model uncertainty by county by computing the entropy, *H*, [52]:

H=−p*log2(p)−(1−p)*log2(1−p) (1)

where *log2* is the base 2 logarithm, and *p* is the county-level ensemble model probability. If all of the candidate models predict absence and have high NPV or if the models predict presence and have high PPV, the ensemble model probability *p* will be close to 0 or 1, respectively, and *H* will be close to 0. If the candidate models disagree or have low PPV or NPV, the ensemble model probability will be close to 0.5 with *H* close to 1 indicating high uncertainty.

Finally, we calculated the ensemble model residuals by county, *E*, for presence and absence counties by subtracting the ensemble model probability, *p*, from the presence or pseudo-absence record, *x*, as follows:

E=x−p (2)

where *x* = 1 for presence and *x* = 0 for pseudo-absence. In this manner, the maximum residual would be *E* = 1 or *E* = -1. A value of *E* = 1 indicates a prediction of absence when presence has been reported (*p* = 0 and *x* = 1) and a value of *E* = -1 indicates a prediction of presence for a county classified as pseudo-absence (*p* = 1 and *x* = 0). The minimum residual occurs when *p* = *x* (*E* = 0). The residual is a signed measure of the difference between the ensemble model probabilities and the presence or pseudo-absence record. We computed *E* on both the training and the testing datasets.

## Results

### Existing models

**Fig 2:**
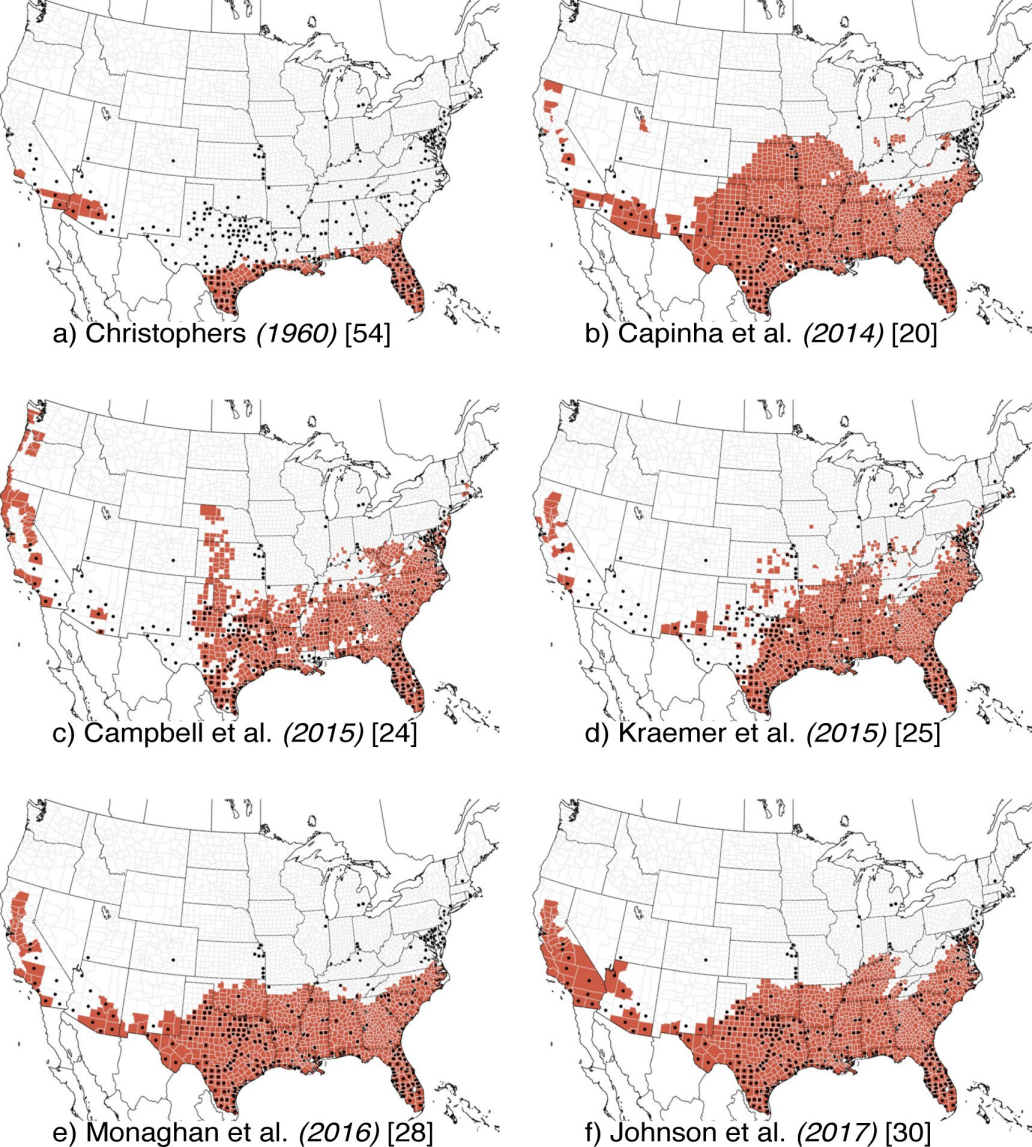
Reproduced *Ae*. *aegypti* models. Distribution models reproduced from (a) Christophers (1960) [54], (b) Capinha et al. (2014) [20], (c) Campbell et al. (2015) [24], (d) Kraemer et al. (2015) [25], (e) Monaghan et al. (2016) [28], and (f) Johnson et al. (2017) [30]. Red areas are predicted to be environmentally suitable for presence. Black dots are all presence records.

**Fig 3:**
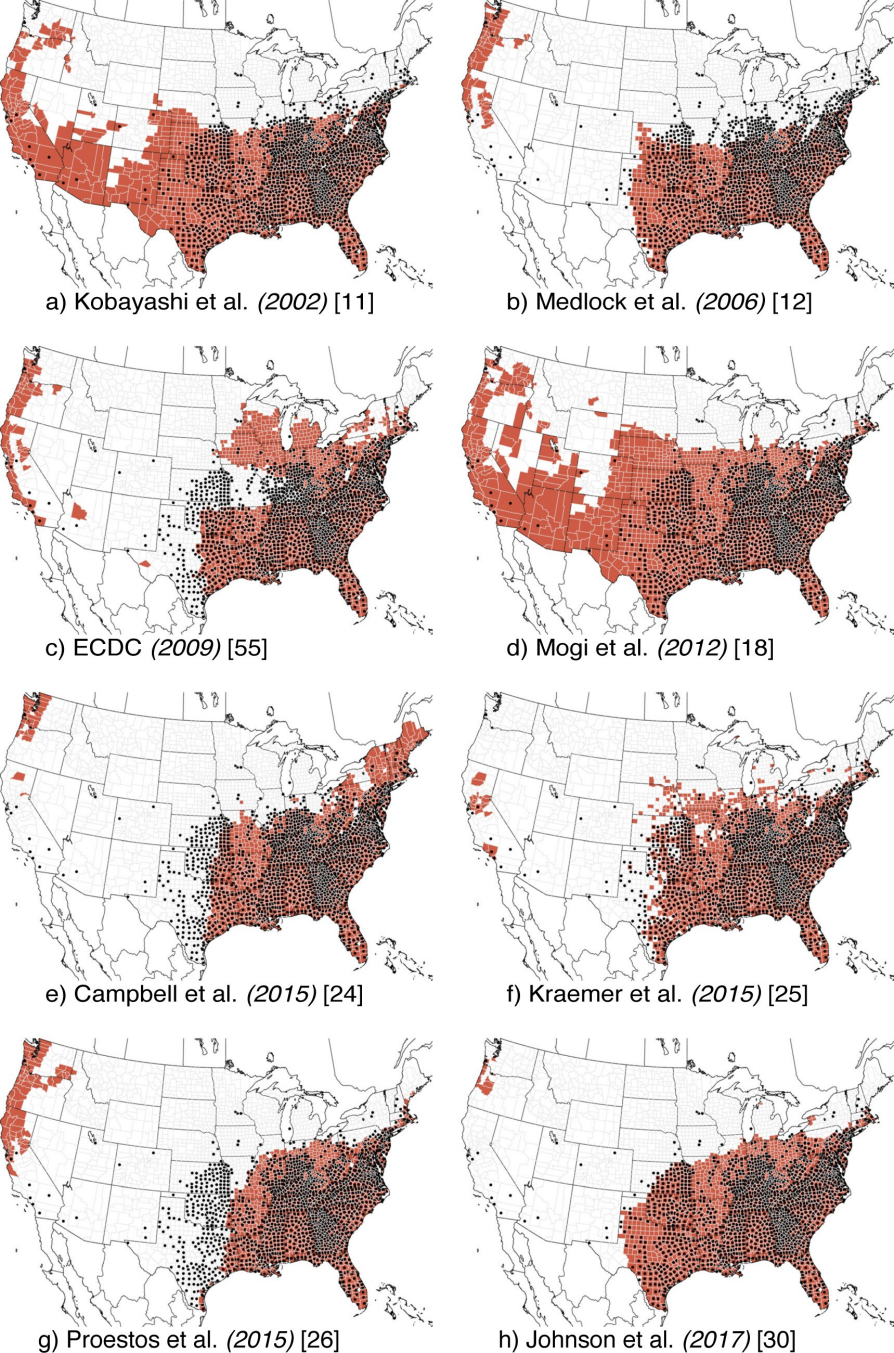
Reproduced *Ae*. *albopictus* models. Distribution models reproduced from (a) Kobayashi et al. (2002) [11], (b) Medlock et al. (2006) [12], (c) ECDC (2009) [55] (d) Mogi et al. (2012) [18], (e) Campbell et al. (2015) [24], (f) Kraemer et al. (2015) [25], (g) Proestos et al. (2015) [26], and (h) Johnson et al. (2017) [30]. Red areas are predicted to be environmentally suitable for presence. Black dots are all presence records.

We evaluated six existing models of the distribution of *Ae*. *aegypti* (Fig 2 and S1 Table) and eight models of the distribution of *Ae*. *albopictus* (Fig 3 and S2 Table) on the training data for each species (233 presence and 565 pseudo-absence counties for *Ae*. *aegypti* and 1254 presence and 155 pseudo-absence counties for *Ae*. *albopictus*). Two of the *Ae*. *aegypti* models [25,30] and three of the *Ae*. *albopictus* models [25,26,30] were converted to binary predictions by identifying the threshold that maximized the average of sensitivity and specificity in the training dataset (S2 Fig). Sensitivity ranged from 0.31 to 0.83 (*Ae*. *aegypti*) and 0.79 to 0.97 (*Ae*. *albopictus*) (Table 1). Specificity ranged from 0.67 to 0.98 (*Ae*. *aegypti*) and 0.56 to 0.98 (*Ae*. *albopictus*). Some models indicated tradeoffs between the two metrics; for example, the Johnson et al. [30] model for *Ae*. *aegypti* had the highest sensitivity but the second lowest specificity. Accuracy ranged from 0.70 to 0.80 for *Ae*. *aegypti*, and was consistently higher for *Ae*. *albopictus*, 0.80 to 0.97. For *Ae*. *aegypti*, the positive predictive values (PPV) were generally low and the negative predictive values (NPV) high, while the opposite was true for *Ae*. *albopictus*.

**Table 1:** Training data fit statistics for the dichotomized base models.

Model (citation)	Sensitivity	Specificity	PPV	NPV	Accuracy
*Ae*. *aegypti*					
Monaghan [28]	0.76	0.81	0.63	0.89	0.80
Christophers [54]	0.31	0.98	0.85	0.77	0.78
Johnson [30]	0.83	0.75	0.57	0.92	0.77
Kraemer [25]	0.74	0.76	0.56	0.88	0.76
Caphina [20]	0.78	0.67	0.49	0.88	0.70
Campbell [24]	0.67	0.71	0.49	0.84	0.70
*Ae*. *albopictus*					
Johnson [30]	0.97	0.98	1.00	0.79	0.97
Mogi [18]	0.98	0.56	0.95	0.80	0.94
Kraemer [25]	0.90	0.94	0.99	0.55	0.91
Kobayashi [11]	0.92	0.64	0.95	0.50	0.89
Campbell [24]	0.84	0.91	0.99	0.41	0.85
Medlock [12]	0.81	0.88	0.98	0.36	0.81
ECDC [55]	0.81	0.77	0.97	0.33	0.80
Proestos [26]	0.79	0.84	0.98	0.34	0.80

### Ensemble model evaluation

**Fig 4:**
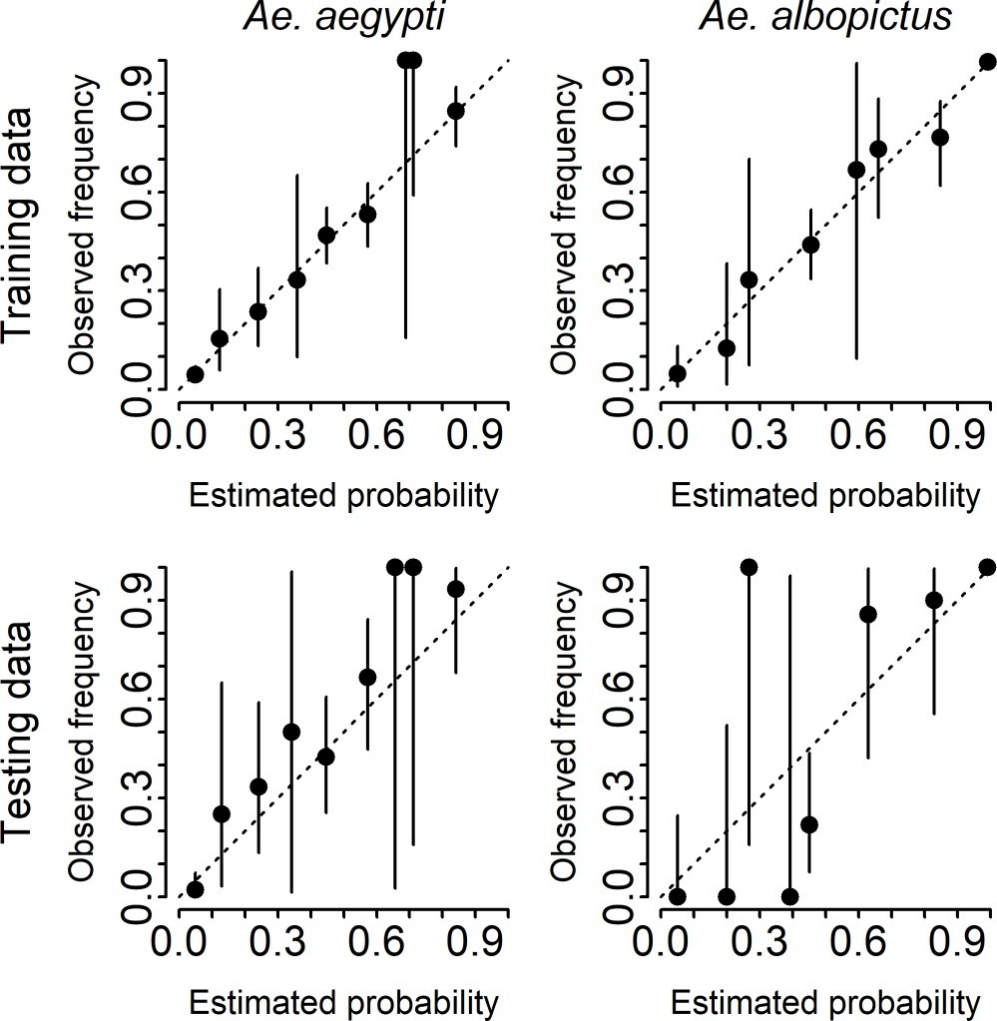
Model calibration. Estimated probability of presence versus the frequency of counties reporting the species as present, by decile, for *Ae*. *aegypti* (left panels) and *Ae*. *albopictus* (right panels). The predictions are compared to both the training data (top panels) and testing data (bottom panels).

We constructed ensemble models for each species using model-specific PPV and NPV for presence and absence predictions, respectively, and calibrated those predictions using a binomial generalized additive model (empirical degrees of freedom of approximately 3.6 and 5.2 for *Ae*. *aegypti* and *Ae*. *albopictus*, respectively). These probabilities were well-calibrated for both the training data (Fig 4, top panels) and the independent testing data (Fig 4, bottom panels), albeit with higher uncertainty due to the smaller sample size (the testing dataset had 58 presence and 141 absence counties for *Ae*. *aegypti* and 314 presence and 39 absence counties for *Ae*. *albopictus*). The 95% confidence intervals for the majority of bins included the expected value.

We compared binary predictions from the ensemble models (probability of presence > = 0.5) to binary predictions from the other models on the independent testing data (Table 2). The ensemble model had the second highest accuracy for *Ae*. *aegypti* (0.83), slightly lower than the Monaghan et al. [28] model (0.85). The ensemble model and the Johnson et al. [30] model both had the highest accuracy for *Ae*. *albopictus* (0.97).

**Table 2:** Testing data fit statistics for the dichotomized base and ensemble models.

Model (citation)	Sensitivity	Specificity	PPV	NPV	Accuracy
*Ae*. *aegypti*					
Monaghan [28]	0.78	0.88	0.73	0.91	0.85
Ensemble [n/a]	0.57	0.94	0.79	0.84	0.83
Johnson [30]	0.81	0.82	0.64	0.91	0.81
Christophers [54]	0.34	0.99	0.95	0.79	0.80
Kraemer [25]	0.76	0.77	0.58	0.89	0.77
Capinha [20]	0.83	0.72	0.55	0.91	0.75
Campbell [24]	0.64	0.74	0.51	0.83	0.71
*Ae*. *albopictus*					
Johnson [30]	0.97	0.97	1.00	0.83	0.97
Ensemble [n/a]	0.98	0.95	0.99	0.84	0.97
Mogi [18]	0.98	0.51	0.94	0.80	0.93
Kraemer [25]	0.92	0.92	0.99	0.58	0.92
Kobayashi [11]	0.92	0.72	0.96	0.53	0.90
Campbell [24]	0.84	0.97	1.00	0.43	0.86
Proestos [26]	0.81	0.90	0.98	0.36	0.82
ECDC [55]	0.81	0.82	0.97	0.35	0.81
Medlock [12]	0.78	0.95	0.99	0.35	0.80

**Fig 5:**
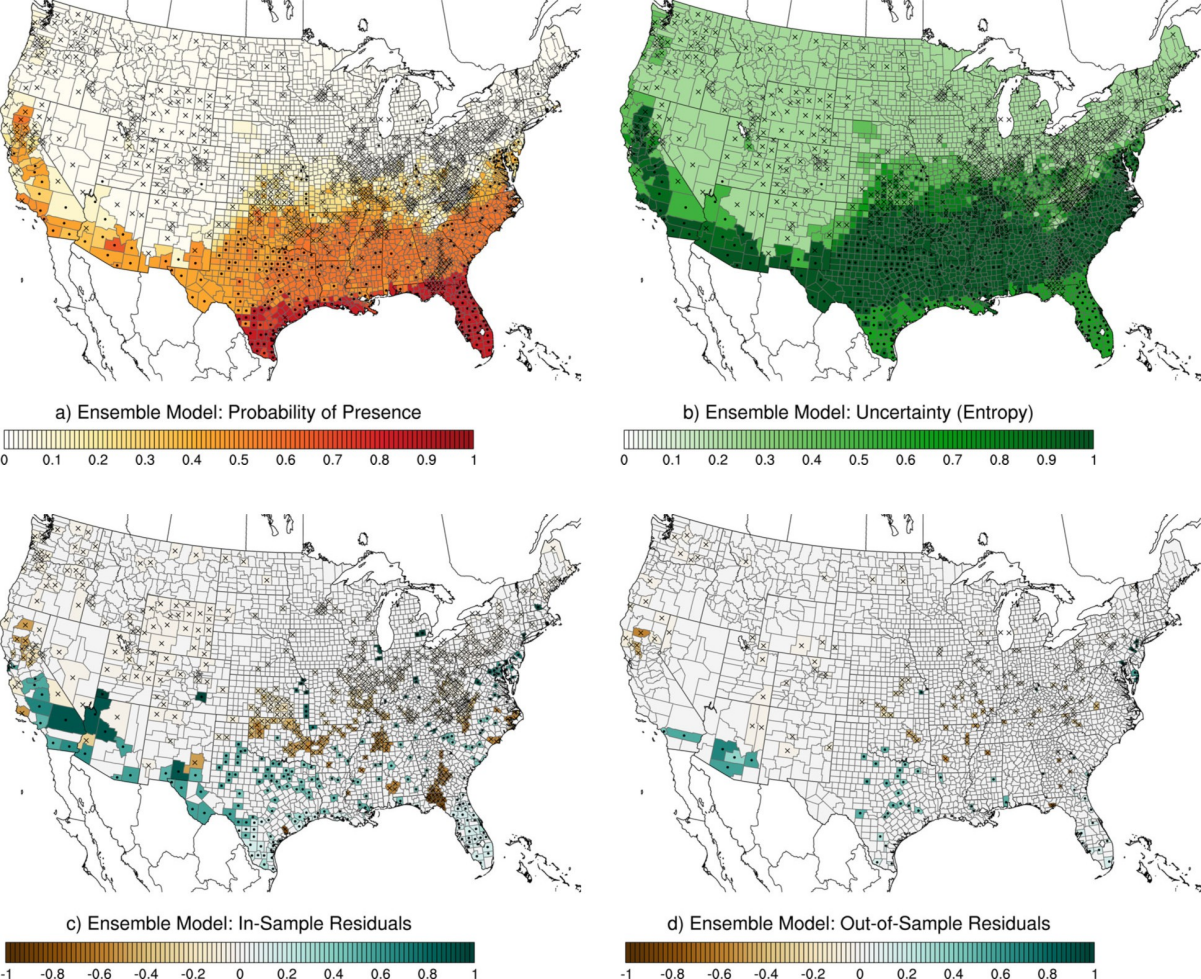
Ensemble model for *Ae*. *aegypti*. (a) Ensemble model probability of presence; (b) ensemble model uncertainty expressed as entropy, *H*; (c) ensemble model in-sample residuals, *E*; and (d) ensemble model out-of-sample residuals, *E*. Black dots are presence records and black "x" marks are pseudo-absence records. All presence and absence records are shown in (a) and (b); only in-sample records are shown in (c); and only out-of-sample records are shown in (d).

For *Ae*. *aegypti*, the ensemble model predicted high probability of presence in Florida and the Gulf Coast to southeastern Texas, with some areas of elevated probability in Arizona and California (Fig 5A). The regions of high uncertainty were extensive, spanning areas with probabilities of approximately 0.4–0.6, including California, southern Arizona, and most of the southeast, from Texas, Oklahoma, and Kansas across to the East Coast from Georgia to New Jersey (Fig 5B). Prediction residuals for the training and testing data exhibited similar patterns (Fig 5C and 5D), with high probabilities for *Ae*. *aegypti* in several areas where the mosquito was not recorded (e.g. parts of northern Florida and southern Georgia and some counties in Oklahoma, Arkansas, Tennessee, Mississippi, Alabama, and North Carolina). The ensemble model predicted low probabilities in numerous counties in California, Arizona, Texas, and Maryland where *Ae*. *aegypti* had been recorded. Removing the 100km buffer to exclude pseudo-absence counties that bordered presence counties resulted in qualitatively similar results, with general lower probability of presence (S3 Fig).

**Fig 6:**
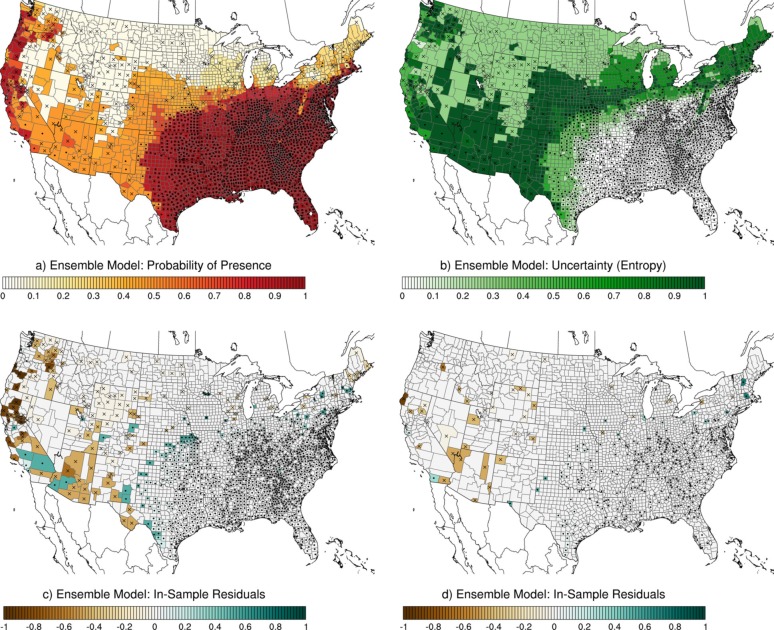
Ensemble model for *Ae*. *albopictus*. (a) Ensemble model probability of presence; (b) ensemble model uncertainty expressed as entropy, *H*; (c) ensemble model in-sample residuals, *E*; and (d) ensemble model out-of-sample residuals, *E*. Black dots are presence records and black "x" marks are pseudo-absence records. All presence and absence records are shown in (a) and (b); only in-sample records are shown in (c); and only out-of-sample records are shown in (d).

The ensemble model predicted a much broader distribution for *Ae*. *albopictus*, with high probabilities of presence throughout the southeast U.S. and along most of the West Coast (Fig 6A). The western limit of probabilities greater than 0.5 for this region was eastern Texas and Oklahoma and the northern limit included Arkansas, southern areas of Illinois, Indiana, Ohio, West Virginia, Maryland, and along the coast up to southwest Connecticut. Beyond this area of high probability, there was a substantial band of high uncertainty that extended south of the Rocky Mountains through New Mexico and Arizona, all the way up the West Coast (Fig 6B). In some of these highly uncertain areas (e.g. west Texas, Oklahoma, and Kansas), many counties had reported *Ae*. *albopictus*, indicating that the ensemble model under-estimated the probability of presence in those areas (Fig 6C and 6D). Further west, through Arizona, California, and further north along the coast, there were more areas where the ensemble model predicted high probabilities of presence but *Ae*. *albopictus* had not been recorded.

## Discussion

Building on extensive previous work to estimate the local or global distributions of *Ae*. *aegypti* and *Ae*. *albopictus*, we developed ensemble models predicting the county-level probabilities of presence of those key vector species in the contiguous U.S. Through a two-stage process, we assessed out-of-sample performance and identified areas with high uncertainty and residual bias that, if targeted for enhanced surveillance activities, may be most beneficial for improving our knowledge of where *Ae*. *aegypti* and *Ae*. *albopictus* mosquitoes are present.

First, in contrast to the original models, we developed a dataset with specific pseudo-absence points defined as counties that had not reported the species despite surveillance efforts that might have detected it. This allowed model comparison for a number of standard accuracy metrics. Across models, newer models tended to perform better, possibly reflecting improved analytical approaches but also likely due to increased data availability. Accuracy and positive predictive values were highest for *Ae*. *albopictus*, possibly related to having more presence data points with which to fit the models or possibly because the surveillance records were more geographically homogenous and may therefore be easier to classify with a model. In contrast, *Ae*. *aegypti* had more pseudo-absence counties than presence counties and the presence counties were more dispersed, contributing to higher negative predictive values, lower positive predictive values, and lower overall accuracy. The dispersal of *Ae*. *aegypti* presence records in the Southeast in particular suggests either high population fragmentation or limited surveillance.

The evaluation of model accuracy and uncertainty suggests that collecting additional surveillance data would enhance efforts to map both species, an effort that is underway [56]. More importantly, our results highlight areas where focused surveillance efforts would likely improve both data and models. The accuracy-weighted ensemble models for each species identified large areas where the models had high uncertainty (entropy). This uncertainty arises from disagreement among models with similar accuracy while certainty comes from agreement across accurate models (inaccurate models are down-weighted). The uncertainty thus highlights areas where either models are failing to capture risk or data are lacking. Specifically, the models highlighted uncertainty in the regions of the South situated north of the Gulf Coast, the Southwest, and California for *Ae*. *aegypti* and the Northeast, the upper Midwest (40°-45°N), the central and southern Great Plains, the Southwest and the entire West Coast for *Ae*. *albopictus*. Areas where uncertainty was high for both species were generally semi-arid and arid regions including western Texas, southern New Mexico and Arizona, and most of California.

Overall, the results are largely in agreement with several of the more recent models, but provide additional probabilistic insight on the areas where data and models are lacking. Some uncertainty is expected due to the limitations of the data. For example, a county where presence is highly unlikely may have a single surveillance record due to an imported mosquito or a county with an established cryptic population may have no records because surveillance has yet to detect the mosquito species. These conditions–low probability with reports of the vector or high probability with no records–are most likely to occur in areas of borderline suitability along the margins of the range of either mosquito (areas with high uncertainty in the ensemble model) or in areas where the distributions may have changed over time.

Despite limitations in the data, predictions on both training and testing data revealed areas with systematic bias in the ensemble residuals. In the Southwest, the probability of presence was low for *Ae*. *aegypti* in numerous counties where the species had been reported but high for *Ae*. *albopictus* in counties where *Ae*. *albopictus* had not been reported, indicating challenges across models in capturing the distributions in this region, possibly due to the arid climate or more recent introductions. In Texas, *Ae*. *aegypti* was more common in the data than predicted while in Oklahoma and Kansas the opposite was true, indicating a possible range limit that is not resolved well by the existing models. Possibly models relying on only environmental data under-estimate the availability of *Ae*. *aegypti* larval habitats created by human water storage in arid areas [57]. For many counties in these three states and along much of the northern boundary of the estimated *Ae*. *albopictus* range, *Ae*. *albopictus* was more common than estimated. This may be a sign of a failure to capture dynamic change, as *Ae*. *albopictus* expansion has been recent [40] and likely is still ongoing. In contrast, *Ae*. *aegypti* predictions assigned high probability to numerous counties along its estimated northern boundary where the species had not been reported. This also occurred for a contiguous patch of counties from northern Florida near Tallahassee into Georgia where *Ae*. *aegypti* have never been reported. In areas where counties with presence and pseudo-absence records are interspersed, such as for *Ae*. *aegypti* in the Southeast, it may be particularly difficult for models to identify the key characteristics that differentiate them.

Some models may hold clues to better characterizing these regions. For example, the models of Johnson et al. [30] and Kraemer et al. [25] captured the western and northern bounds of the southeastern *Ae*. *albopictus* population fairly well despite lower accuracy in the Southwest. These two particular models were trained with some of the most comprehensive surveillance datasets among all models. However, it is also possible that the most important ancillary determinants of presence have yet to be identified. For example, all included models used macroclimatic data, yet micro climatic variation and human factors also play an important role [58] (e.g. water storage practices, mosquito control practices, human population density).

Comparing the accuracy of binary predictions on the testing dataset, we found that the ensemble model was the second most accurate model for *Ae*. *aegypti* and matched the Johnson et al. [30] model for highest accuracy for *Ae*. *albopictus*. However, this comparison is a simplified indicator of accuracy, because most models did not provide probabilistic predictions, necessitating comparison on a binary scale (i.e. presence or absence). Notably, those models that did include non-binary predictions did not generally appear to be well calibrated in the sense that their cutoff probabilities for presence/absence were typically much greater- or less-than the expected value of 0.5 (S2 Fig). The ensemble model, on the other hand, provides calibrated probabilistic predictions, such that a prediction of 0.5 indicates a 50% chance of presence. For example, the ensemble model shows that presence of *Ae*. *aegypti* north of the Gulf Coast states is not 100% certain and presence in the Chesapeake Bay area is a distinct possibility. Probabilistic forecasts also allow more detailed assessment of residuals, as discussed above.

Our analysis revealed some new insights, but also had important limitations. First, we relied on a limited set of data collected over several decades. Resolving dynamic changes over time is important to understanding present-day risk and supporting seasonal vector control planning, but trapping is highly resource intensive and large-scale, longitudinal data are particularly limited. Collecting data at broad spatiotemporal scales is an intrinsic challenge for this type of analysis. Even where there are surveillance data, those data are inherently limited by the collection technique used (e.g., type of trap and manner in which it is deployed) and approaches and efforts are highly varied [39]. Here, we addressed the lack of true absence data by incorporating more specific indicators of absence than previous studies, but they are still imperfect. For example, in counties where *Ae*. *aegypti* was classified as pseudo-absent because mosquito surveillance was reported or *Ae*. *albopictus* had been recorded there but *Ae*. *aegypti* had not, we intrinsically assumed that trapping methods would be suitable for both species. However, it may be that some traps were placed in sparsely populated rural areas more likely to be inhabited by *Ae*. *albopictus* than *Ae*. *aegypti* [51]. In addition to the possibility of mis-categorizing absence, false positives for presence are also possible due to misidentification, adventitious mosquitoes, or transitory establishment. The challenge of classifying both absence and presence also impacts interpretation of the outcome. The probabilistic ensemble models developed here represent an advance in estimating presence because they were weighted and calibrated to presence and more specific absence data than previous studies, yet these underlying challenges persist.

Another significant challenge was collecting and reproducing previously published models. Some publications did not contain sufficient information to reproduce the models and the majority of those that did only allowed reproduction of binary predictions (presence or absence), despite underlying probabilistic models. We therefore assessed accuracy and developed the ensemble model based on dichotomized versions of all models rather than richer probabilistic predictions. Ideally, all models should publish and assess predictions as probabilities.

*Ae*. *aegypti* and *Ae*. *albopictus* are important well beyond CONUS. In many tropical areas, the vectors are ubiquitous, but in others regions they are geographically limited, particularly at more extreme latitudes and higher altitudes. In most regions, the data are even more sparse, exacerbating the challenges presented here but not diminishing the importance of quantitative out-of-sample assessment and synthesis of previous model outputs in calibrated probabilistic estimates, key components that are even more important with less robust data. The framework developed here should be more broadly employed to identify the dynamic geographical ranges of these species. Better characterizing these vector distributions, or even just their uncertainty, in CONUS and beyond can guide resources for implementing surveillance and control efforts to minimize risk.

Moreover, the distributions of these species are just examples from much broader mapping efforts to help prepare and respond to infectious disease threats. The framework for evaluation presented here can serve as a model for aggregating and assessing information. First, models should be reproducible and should include probabilistic outputs. Evaluations should characterize uncertainty, calibration, and bias, the latter two on out-of-sample data. These analyses are missing in the majority of published maps characterizing vectors and other aspects of infectious disease risk. Only by validating probabilistic predictions on out-of-sample data can we characterize the strengths, weaknesses, and reliability of models. Understanding these characteristics is critical both for improving models and risk estimates and for their intended use, to improve decision-making and protect health.

## Supporting information

S1 Fig Counties with partial or full surveillance coverage for mosquitoes as of 28 April 2017. Counties identified as conducting partial or full surveillance for mosquitoes as of 28 April 2017 (compiled by JM from multiple sources).
(EPS)

S2 Fig Sensitivity, specificity and 'adjusted accuracy' of non-dichotomous models. Sensitivity, specificity and 'adjusted accuracy' (the average of sensitivity and specificity) versus cutoff probability to dichotomize the following models: (a) Kraemer et al. (2015), Ae. aegpti; (b) Johnson et al. (2017), Ae. aegypti; (c) Kraemer et al. (2015), Ae. albopictus; (d) Proestos et al. (2015), Ae. albopictus; and (e) Johnson et al. (2017), Ae. albopictus. The cutoff value with the highest adjusted accuracy (indicated in the X-axis label) was used to dicohomize estimates from these models.
(EPS)

S3 Fig Comparison of ensemble predictions with and without the 100km buffer. Ensemble model probability of presence for *Ae*. *aegypti* computed using (a) all psuedo-absence records or (b) including only pseudo-absence counties that were at least 100 km away from a presence county. Ensemble model probability of presence for *Ae*. *albopictus* computed using (c) all psuedo-absence records or (d) pseudo-absence counties with the 100 km buffer. Probabilities were not calibrated, but for ease of comparison were rescaled such that the minimum is 0 and the maximum is 1.
(EPS)

S1 Table Final set of candidate models used for *Ae*. *aegypti* model synthesis. Descriptions of included individual suitability models for *Ae*. *aegypti*.
(DOCX)

S2 Table Final set of candidate models used for *Ae*. *albopictus* model synthesis. Descriptions of included individual suitability models for *Ae*. *albopictus*.
(DOCX)

S1 Data Data for the *Ae*. *aegypti* models and figures. Spreadsheet including columns for county FIPS codes, names, latitude, longitude, inclusion in the training/testing datasets as present (1) or pseudo-absent (0), model-specific binary predictions, and calibrated probabilistic ensemble predictions.
(CSV)

S2 Data Data for the *Ae*. *albopictus* models and figures. Spreadsheet including columns for county FIPS codes, names, latitude, longitude, inclusion in the training/testing datasets as present (1) or pseudo-absent (0), model-specific binary predictions, and calibrated probabilistic ensemble predictions.
(CSV)
