# Supplementary material for: Consensus and uncertainty in the geographic range of Aedes aegypti and Aedes albopictus in the contiguous United States: Multi-model assessment and synthesis
Source: PLoS Comput Biol. 2019 Oct 10;15(10):e1007369. doi: 10.1371/journal.pcbi.1007369 (PMC6786520; doi:10.1371/journal.pcbi.1007369)
Supplement: S1 Table — Descriptions of included individual suitability models for Ae. aegypti. (DOCX) [file pcbi.1007369.s004.docx]

**S1 Table.** Final set of candidate models used for *Ae. aegypti* model synthesis.

**S2 Table.** Final set of candidate models used for *Ae. albopictus* model synthesis.
